# Acidic Residues Control the Dimerization of the N-terminal Domain of Black Widow Spiders’ Major Ampullate Spidroin 1

**DOI:** 10.1038/srep34442

**Published:** 2016-09-29

**Authors:** Joschka Bauer, Daniel Schaal, Lukas Eisoldt, Kristian Schweimer, Stephan Schwarzinger, Thomas Scheibel

**Affiliations:** 1 Lehrstuhl Biomaterialien, Universität Bayreuth, Fakultät für Ingenieurswissenschaften, Bayreuth, Germany; 2 Lehrstuhl Biopolymere, Universität Bayreuth, Bayrisches Geoinstitut, Bayreuth, Germany; 3 Forschungszentrum für Bio-Makromoleküle (BIOmac), Universität Bayreuth, Bayreuth, Germany; 4 Bayreuther Zentrum für Kolloide und Grenzflächen (BZKG), Universität Bayreuth, Bayreuth, Germany; 5 Bayreuther Materialzentrum (BayMat), Universität Bayreuth, Bayreuth, Germany; 6 Bayreuther Zentrum für Molekulare Biowissenschaften (BZMB), Universität Bayreuth, Bayreuth, Germany

## Abstract

Dragline silk is the most prominent amongst spider silks and comprises two types of major ampullate spidroins (MaSp) differing in their proline content. In the natural spinning process, the conversion of soluble MaSp into a tough fiber is, amongst other factors, triggered by dimerization and conformational switching of their helical amino-terminal domains (NRN). Both processes are induced by protonation of acidic residues upon acidification along the spinning duct. Here, the structure and monomer-dimer-equilibrium of the domain NRN1 of *Latrodectus hesperus* MaSp1 and variants thereof have been investigated, and the key residues for both could be identified. Changes in ionic composition and strength within the spinning duct enable electrostatic interactions between the acidic and basic pole of two monomers which prearrange into an antiparallel dimer. Upon naturally occurring acidification this dimer is stabilized by protonation of residue E114. A conformational change is independently triggered by protonation of clustered acidic residues (D39, E76, E81). Such step-by-step mechanism allows a controlled spidroin assembly in a pH- and salt sensitive manner, preventing premature aggregation of spider silk proteins in the gland and at the same time ensuring fast and efficient dimer formation and stabilization on demand in the spinning duct.

Spider dragline silk is used by orb weavers as a lifeline to escape from predators and as a stabilizing frame in their webs due to its excellent mechanical properties including an outstanding toughness. It comprises proteins produced in the major ampullate silk gland classified as major ampullate spidroins MaSp1^1^ and MaSp2^2^ dependent on their amino acid composition, mainly their proline content which is low (<1%) in MaSp1 and high (~9%) in MaSp2^3^. Spidroins are stored in a soluble form at high concentrations (>30% w/v) in the presence of NaCl in the lumen of the silk gland^4^,^5^, and fiber assembly is initiated upon passage of the spidroin solution through the spinning duct^6^. The pH decreases along the duct from pH 7.2 to 6.0 or below, depending on the spider species, and sodium chloride is replaced by potassium phosphate, both of which together with shear forces induce spidroin assembly^5^,^7^,^8^,^9^,^10^,^11^. Within the spinning dope, the large repetitive MaSp core domains (>1000 amino acids) are assumed to be intrinsically disordered, while the short (~100–150 amino acids) non-repetitive N- (NRN) and C-terminal domains (NRC) exhibit well defined α-helical structures^3^,^12^,^13^. These terminal domains control spidroin solubility in the dope and initiate fiber assembly upon passage through the spinning duct. Fiber assembly is accompanied by conversion of the intrinsically disordered core domain into a β-sheet-rich (~25–40%) one^14^,^15^,^16^,^17^,^18^,^19^. Due to their important function during assembly, the sequences and therefore the three-dimensional fold of NRC and especially NRN are highly conserved, not only of individual silks between spider species, but also between different silk types within one species^20^,^21^,^22^,^23^,^24^. While NRC of most MaSp are permanent disulphide-linked dimers even under storage conditions in the gland, dimerization of NRN is triggered in a pH and salt dependent manner within the duct^25^,^26^,^27^. The previously determined structure of *Euprosthenops australis* (NRN1_*E.a.*_) as well as of *Latrodectus hesperus* (NRN1_*L.h.*_) MaSp1 revealed a dipolar charge distribution, with charged amino acids being grouped into a basic NH_2_- and an acidic COOH-terminal pole^26^,^28^,^29^. In NRN1_*E.a.*_, an acidic cluster, comprising at least six surface exposed aspartic and glutamic acid residues, has been assumed to mediate a pH-dependent dimerization upon conversion from a dimer-incompatible into a dimer-favouring conformation^28^. The monomeric wildtype (wt) structure, denoted as conformation I, is stabilized both in the presence of sodium chloride and at a neutral pH (7.2). Acidification triggers the formation of conformation II upon dimerization. Previously, the conformational change was monitored by a bathochromic shift of the fluorescence emission of a single, naturally occurring Trp residue (W9) indicating its relocation into a more hydrophilic environment on the surface^12^,^28^. Underlining their likely importance for this conformational change, one aspartic acid (D39) and three glutamic acid residues (E76, E81, E114) are the most conserved residues within NRN among all spider species so far investigated^20^,^21^,^22^,^23^,^24^.

Here, the dimerization and conformational changes of the amino-terminal domain of *Latrodectus hesperus* MaSp1 (NRN1_*L.h.*_) have been investigated in more detail. A variety of different techniques such as tryptophan fluorescence, near- and far-UV circular dichroism, multi-angle light scattering as well as two-dimensional NMR-spectroscopy were applied to characterize wtNRN1_*L.h.*_and variants thereof. The role of the most conserved acidic residues was identified by replacing them by their non-titratable analogues asparagine and glutamine to mimic the protonated state caused by acidification within the spinning duct. While protonation of the single side chain E114 upon acidification is sufficient to trigger dimer formation, the structural conversion into the final dimer conformation is mediated by the acidic cluster. Charged residues D39 and E81 prearrange the antiparallel dimer by electrostatic interactions with basic residues of the second subunit. Dimerization and structural rearrangement occur independently and are controlled by separated regions of the domain.

## Materials and Methods

### Protein production

Genes encoding the amino-terminal domain of *Latrodectus hesperus* major ampullate spidroin 1 were cloned into pET28a, containing a sequence encoding a His6-SUMO-tag (Novagen). Mutations were introduced using a QuikChange^®^ Site-Directed Mutagenesis Kit (Agilent Technologies). Genes were expressed in *Escherichia coli* strain BL21 (DE3) (Agilent Technologies). The cells were grown to an OD_600_ = 8 in ZYM-5052 autoinduction medium^30^ containing kanamycin for 17 h at 30 °C. Proteins were purified by nickel-NTA chromatography (HisTrap FF, GE Healthcare) and size exclusion chromatography (HiLoad™ 26/60 Superdex™ 75 pg, GE Healthcare). The His6-SUMO-tag was cleaved off by addition of a SUMO-protease and incubation for 1 h at RT. SUMO-tag and protease were separated from the spidroin using a second nickel-NTA chromatography step.

### Fluorescence spectroscopy

Tryptophan fluorescence was analysed using a Jasco FP-6500 spectrofluorometer with a 3 mm path length. The spectra were recorded at an excitation wavelength of 295 nm and an emission wavelength between 300 and 450 nm. The pH-titration was performed by successively adding 5 μl of 0.1 M H_3_PO_4_ to 1 ml of protein solution (14.2 μM).

### CD spectroscopy

Circular dichroism spectroscopy measurements were performed using a Jasco J-715 spectropolarimeter. CD spectra were acquired at a response time of 1 s, a scanning speed of 50 nm min^−1^ and a bandwidth of 1 nm. Far-UV CD spectra and thermal transitions were measured at 14.2 μM using a path length of 0.1 cm. Thermal transitions were recorded at a wavelength of 222 nm and a heating rate of 60 °C h^−1^. Near-UV CD spectra were monitored at protein concentrations of 35.5 μM and 142.5 μM (Supplementary Fig. S1) using a path length of 0.5 cm, and, respectively, of 1.0 cm. Near-UV CD spectra were processed by applying a Savitzky-Golay filter^31^.

### SEC-MALS

Size-exclusion chromatography (SEC, Superdex™ 200 10/300 GL, GE Healthcare) was performed on an Agilent 1100 system to pre-separate the protein solution for the subsequent analysis. The flow rate of 20 mM sodium phosphate buffer (pH 7.2 or 6.0 ± 300 mM NaCl) was set to 0.7 ml min^−1^, and 250 μl of protein solution (71.2 μM) were injected at 0.2 ml min^−1^. Multi-angle light scattering (MALS) and quasi-elastic light scattering (QELS, WYATT) were employed to determine the molecular weight (MW) and, thus, the monomer-dimer equilibrium of NRN. The data were evaluated using the ASTRA software (WYATT).

### NMR analysis

Isotopically enriched proteins were produced in the *E. coli* strain BL21 (DE3) grown in M9 minimal medium supplemented with ^15^N ammonium sulphate for ^15^N HSQC and additionally with ^13^C glucose for triple resonance experiments. The cells were grown to an OD_600_ = 4 at 37 °C, and genes were expressed upon addition of 1 mM IPTG for 4 h. All variants were purified in the same manner as their unlabelled analogues. 10% (v/v) D_2_O was added to NMR samples in 22 mM sodium phosphate, and the pH was carefully adjusted to either 7.2 or 6.0. The pH-induced conformational change of triple mutant 3*, in which the acidic cluster was neutralized (D39N-E76Q-E81Q), shifted from pH 6.5 in wtNRN1 to 6.0 as seen in fluorescence titration experiments. Therefore, the respective HSQC experiment was recorded at pH 5.5 instead of 6.0 to ensure complete structural conversion.

All NMR spectra were recorded on a Bruker Avance 700 MHz NMR spectrometer equipped with a 5 mm TCI cryogenic and a TXI probe with Z-axis gradients, respectively. The ^15^N HSQC experiments were recorded according to Mori *et al*.^32^. *T*_1_ and *T*_2_^15^N-relaxation data were recorded for the determination of the oligomerization state of E114Q at pH 7.2. The intensities of the HSQC-type spectra were fitted to mono-exponential decays using the program curve fit (Palmer, Dept. of Biochemistry and Molecular Biophysics, Columbia University, USA). The rotational correlation time *τ*_c_ was estimated using the equation


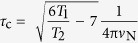


with *T*_1_ and *T*_2_ as the longitudinal and transversal relaxation times and *ʋ*_N_ as the ^15^N nuclear frequency, respectively^33^. Subsequently, the molecular weight was approximated by extrapolation using a set of standard proteins^34^.

High resolution structural data (PDB: 2N3E) of variant 3* were obtained by standard triple-resonance experiments and resonance assignment as described in detail in Schaal *et al*.^29^. The structure of 3* is used here as a structural template to highlight the positions of the most important residues. A structural comparison to the previously published^26^ wtNRN1_*L.h.*_ was not possible since the wildtype structure has not been deposited in the pdb databank (www.rcsb.org)^35^. Distance restraints were collected from NOESY spectra and roughly classified from very strong to very weak (2.7 to 5.5 Å) dependent on their spectral signal intensity. 120 structures were calculated using simulated annealing protocols of the XPLOR-NIH (1.2.1) software package^36^,^37^. Statistics, energetic and structural analysis were performed on the twenty lowest energy structures (Supplementary Fig. S2) using in-house software and PROCHECK^38^,^39^. Structural statistics are summarized in Supplementary Table S1.

NMR spectra were analysed and NMR distance restraints were collected using the CCPNMR software package^40^. HSQC images were generated by NMRViewJ (Newmoon Scientific, Westfield, NJ, USA). Images and structural alignment of 3* were made using MacPyMOL^41^. The final editing of all images was performed using Adobe Illustrator CS3 (Adobe, San Jose, CA, USA).

## Results

### Designing NRN1_*L.h.*_ variants of MaSp1 from the black widow spider with modified charge distribution

**Figure 1:**
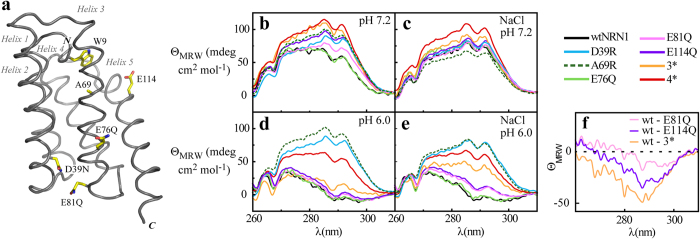
Structural analysis of wildtype and variants of NRN1_*L.h*_. (**a**) The lowest energy structure of the NRN1_*L.h.*_ variant 3* was solved by NMR spectroscopy and is used as a structural template to show the position of the individually mutated amino acid residues (PDB accession code: 2N3E). **(b**–**e**) Near-UV CD spectra of wtNRN1_*L.h.*_ and variants thereof at (**b**) pH 7.2, **(c)** pH 7.2 in the presence of 300 mM NaCl, (**d**) pH 6.0, **(e)** pH 6.0 in the presence of 300 mM NaCl. (**f**) The near-UV CD spectra of E81Q, E114Q and 3* were subtracted by the spectrum of wtNRN1_*L.h.*_to illustrate the significant spectral differences at pH 7.2. All spectra were taken at 142.5 μM.

Throughout 34 so far analysed sequences of amino-terminal domains from different types of spidroins or spider species, amino acid residues D39, E76, E81 and E114 are the most conserved ones^24^. These residues were replaced in the amino-terminal domain (NRN1_*L.h.*_) of MaSp1 of the black widow spider (*Latrodectus hesperus*) by the non-titratable analogues asparagine and glutamine (Fig. 1a) to investigate their contribution to dimerization and conformational conversion. Four single (D39N, E76Q, E81Q, E114Q), one triple (D39N-E76Q-E81Q = 3*) and one quadruple mutant (D39N-E76Q-E81Q-E114Q = 4*) were investigated using several independent methods. In addition, effects of charge reversal (D39R) and charge insertion (A69R) were analysed.

### Identification of key residues of the pH- and salt-induced structural conversion

The pH-dependent conformational state of wtNRN1_*L.h.*_was determined by far- and near-UV circular dichroism (CD) spectroscopy revealing a significant pH-dependence of tertiary (Fig. 1b–e) but not of secondary structure (Supplementary Fig. S3). Mutating individually E81 or E114 clearly affected the environment of the aromatic amino acid side chains and particularly that of the single Trp (W9), and the effect was even more pronounced in the triple mutant 3* (D39N-E76Q-E81Q) (Fig. 1b,f). Hence, negative charges appeared crucial for stabilizing the monomeric conformation I at neutral pH. Starting from wtNRN1_*L.h.*_in conformation I, the five-helix bundle rearranged upon acidification into conformation II, thereby shifting W9 as indicated by a decreased ellipticity (Fig. 1b,d). Addition of NaCl clearly suppressed the structural conversion of 3* (Supplementary Fig. S4), suggesting a role of the mutated acidic residues as a salt-sensitive switch.

**Figure 2:**
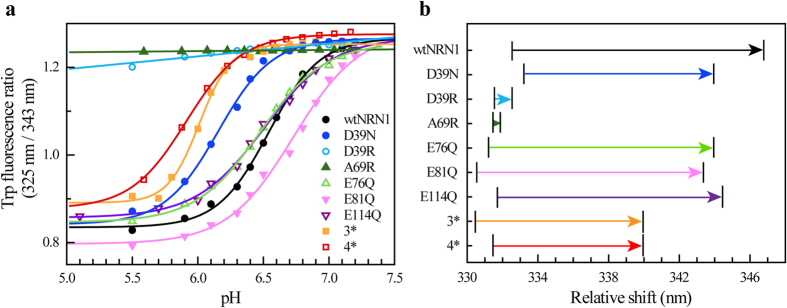
Trp fluorescence analysis of NRN1_*L.h.*_variants. (**a**) The ratio of fluorescence at 328 nm and 343 nm indicates the proportion between conformation I and II. The lines represent fits based on a two-state model. (**b**) Shift of the fluorescence maximum upon lowering the pH from 7.2 to 5.5 (see also Supplementary Fig. S5).

**Figure 3:**
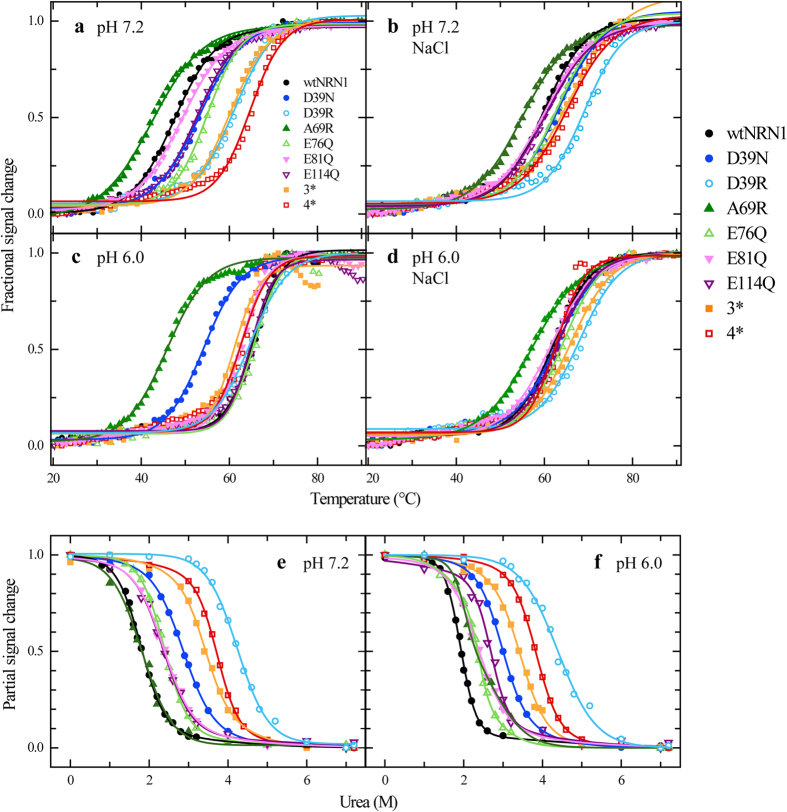
Stability analysis of NRN1_*L.h.*_variants. Protein denaturation was analysed as a function of urea concentration or temperature using CD ellipticity at 222 nm. (**a**–**d**) Thermal denaturation of NRN1_*L.h.*_variants at (**a**) pH 7.2, (**b**) pH 7.2 in the presence of 300 mM NaCl, (**c**) pH 6.0 and (**d**) pH 6.0 in the presence of 300 mM NaCl. **(e,f)** Urea titration of NRN1_*L.h.*_variants at (**e)** pH 7.2 and (**f**) pH 6.0. The lines represent fits using a two-state model.

The importance of charge-charge interactions at the dimerization interface was emphasized by effects of charge-reversal mutations. Introduction of basic residues into the acidic cluster prevented the controlled structural conversion (Fig. 1b,d). Strikingly, the conformational change of wtNRN1_*L.h.*_ involved the relocation of residue W9 into a more hydrophilic environment, which can be seen by a fluorescence redshift with a transition point at around pH 6.5 (Fig. 2a). Neutralization of side chain D39 reduced the fluorescence redshift (Fig. 2b) and lowered the fluorescence transition to pH 6.2 (Fig. 2a). Both effects were even stronger upon deletion of the entire acidic cluster (3*). Accordingly, the local cumulation of residues D39, E76 and E81 elevates their putative p*K*_a_ to values near the physiological pH and enables wtNRN1_*L.h.*_ to completely switch between conformation I and conformation II at slightly acidic pH values (Fig. 2a). Clustering of acidic residues significantly destabilizes the wildtype protein, verified by an increased chemical and thermal stability of D39R (Fig. 3 and Supplementary Table S2). Furthermore, deletion of the acidic cluster clearly reduced the rearrangement into conformation II in the presence of salt (Fig. 1c,e and Supplementary Fig. S4). This finding suggests that the clustered residues D39, E76 and E81 cooperatively act as a pH- and salt-sensitive sensor controlling the structural conversion of NRN1_*L.h*_.

Introduction of an arginine residue within helix 2 (D39R) or helix 3 (A69R) clearly supported conformation I (Figs 1 and 2). Neutralizing E81 or even the entire acidic cluster (3*) considerably altered not only the vicinity of the mutated residues, but the overall conformation I (Fig. 1a,f). Presumably, this is a consequence from lacking electrostatic repulsions between helix 2 and 3. Intramolecular electrostatic repulsions within the acidic cluster are confirmed by an increased protein stability of 3* (Fig. 3 and Supplementary Table S2) and a simultaneously reduced conformational change in the presence of salt (Fig. 1e and Supplementary Fig. S4). The altered conformation I of 3* does not completely prevent conformational changes upon acidification, indicating that additional so far unknown residues might be involved in attaining conformation II.

### The monomer-dimer equilibrium is not affected by structural conversion

**Figure 4:**
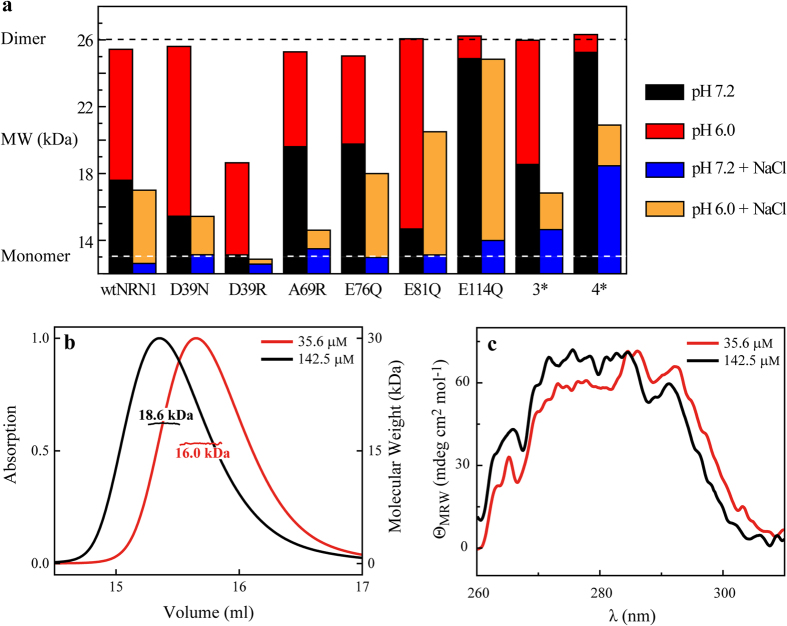
Analysis of monomer-dimer distribution of wildtype as well as variants of NRN1_*L.h.*_. (**a**) Mean molecular weights indicate the ratio between monomers and dimers of NRN1_*L.h.*_variants. MALS of 71.2 μM protein was measured at different pH values in the absence and presence of 300 mM NaCl. (**b**) The normalized UV signal shows the SEC profile of 35.6 μM and 142.5 μM wtNRN1_*L.h.*_at pH 7.2. The molecular weight was determined using MALS. (**c**) Near-UV CD spectra of 35.6 μM and 142.5 μM wtNRN1_*L.h.*_ were taken at pH 7.2.

Monomer-dimer equilibria of the NRN1_*L.h.*_variants were examined using multi-angle light scattering (MALS) analysis. Decreasing the pH from 7.2 to 6.0 triggered wildtype dimerization, whereas the monomeric state was stabilized in the presence of sodium chloride (Fig. 4a and Supplementary Fig. S6). Increasing the wtNRN1_*L.h.*_ concentration slightly shifted the monomer-dimer equilibrium towards the dimer state as expected (Fig. 4b). Near-UV CD spectra revealed that the monomer conformation I is adjusted to a dimer-compatible conformation I* with the Trp (W9) being slightly reoriented (Fig. 4c).

Although the dimerization interface was different due to sterical constraints by replacing a small alanine residue with arginine and its bulky side chain, the monomer-dimer equilibrium of A69R was similar to that of wtNRN1_*L.h*_. The variant A69R showed a wt-like dimerization behaviour but an inhibited structural conversion, indicating independence of both processes upon structurally rearranging NRN1_*L.h.*_ (Figs 1 and 2). Single mutations of residues D39 and E81 into neutral amino acids did not trigger dimerization, but even stabilized the monomeric state at neutral pH (Fig. 4a). Accordingly, the charged side chains of both residues are involved in electrostatic interactions with basic residues of the second subunit at an early stage of dimerization which is necessary to prearrange the subunits in an antiparallel manner. Contrary to the dimerization of NRN1_*E.a.*_^10^,^24^,^28^,^42^, structural information such as Trp fluorescence could not be used to predict the monomer-dimer equilibrium of NRN1_*L.h.*_ since in this case both processes occurred independently.

Variant E114Q was considerably stabilized in the dimeric state at pH 7.2 (Fig. 4a), suggesting that protonation (i.e. neutralization) of residue E114 likely triggered and definitely stabilized dimer formation in a pH-dependent manner. Addition of NaCl-induced disassembly of E114Q dimers, and neutralization of E114 as well as the acidic cluster (variant 4*) reduced such salt-induced dimer disassembly, indicating electrostatic interactions between the acidic and basic pole across the dimerization interface. Variant 4* showed both a slight suppression of dimerization in the presence of NaCl as well as an unaltered conformation (Fig. 1b,c), corroborating that dimerization and structural conversion of NRN1_*L.h.*_are independent and non-related events.

### HSQC fingerprints show that changes in the acidic cluster initiate the conformational reorganisation

**Figure 5:**
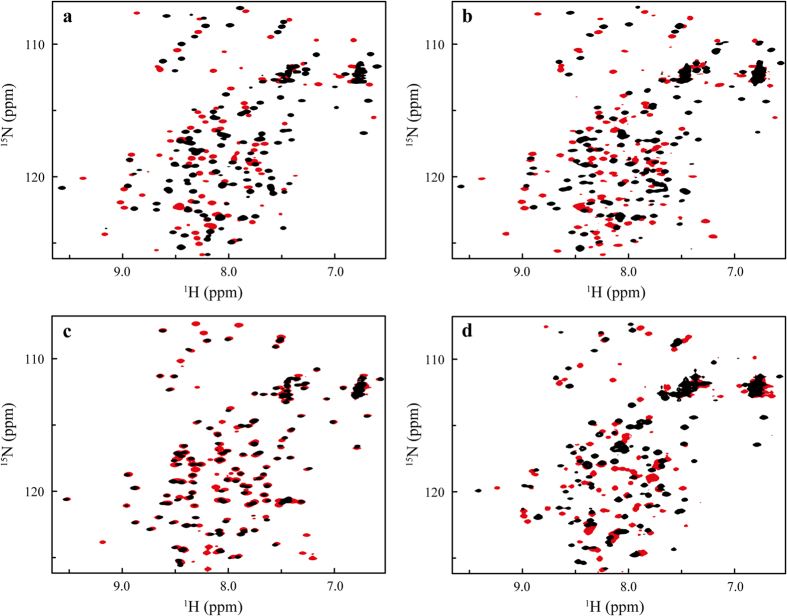
HSQC spectra of wtNRN1_*L.h.*_, E114Q, D39R and 3*. ^15^N-HSQC spectra of (**a**) wtNRN1_*L.h.*_, (**b**) E114Q and (**c**) D39R have been taken at pH 7.2 (black) and pH 6.0 (red). (**d**) Since the conformational change of 3* was shifted to a lower pH, the ^15^N-HSQC spectra of 3* was measured at pH 7.2 (black) and at pH 5.5 (red).

The independence of conformational switching and dimerization was confirmed by comparison of ^15^N-HSQC spectra as well as ^15^N-relaxation experiments. wtNRN1_*L.h.*_showed characteristic ^15^N-HSQC fingerprints for its distinct conformation I at pH 7.2 and conformation II at 6.0, respectively, with severe differences in the chemical shifts (Fig. 5a). In agreement with near-UV CD (Fig. 1), HSQC spectra of E114Q showed a slightly altered conformation I* at pH 7.2 (Supplementary Fig. S7). An NMR relaxation experiment independently indicated dimerization of E114Q at neutral pH in the absence of salt by determining a rotational correlation time *τ*_c_ of 17 ns corresponding to an estimated MW of 27 kDa (Supplementary Fig. S8). This finding provides clear evidence that in the case of NRN1_*L.h.*_, residue E114 (theoretical p*K*_a_ in NRN1_*E.a.*_: 4.4~6.7)^43^,^44^ is the ion/pH-sensitive switch that controls dimerization. However, rearrangement into conformation II is not prevented by charge deletion of E114 (Figs 1, 2 and 5b and Supplementary Fig. S7), showing that the structural conversion is controlled by additional residues and occurs independently from dimer formation.

Reversing the charge of residue D39 of the acidic cluster (D39R) stabilized the wt-like conformation I between pH 7.2 and pH 6.0 (Fig. 5c and Supplementary Fig. S7). Together with unchanged near-UV CD and fluorescence spectra in the same pH-range (Figs 1 and 2) this indicates that the interplay of clustered acidic residue side chains is required for the change into conformation II. Neutralizing three charges of the cluster (3*) induced significant differences as seen in the ^15^N-HSQC spectrum at neutral pH conditions (Fig. 5d and Supplementary Fig. S7). Triple-resonance NMR spectroscopy showed that the overall structure of *L.h.* 3* (Supplementary Fig. S2, pdb 2N3E) resembled the five-helix bundle of NRN1_*E.a.*_^12^,^28^. A detailed triple resonance assignment is given in Schaal *et al*.^29^. In summary it can be assumed that the charges of the acidic cluster are not required to adopt the overall structure of wtNRN1_*L.h.*_, but they significantly affect conformation I. The rearrangement into conformation II seems to be initiated by neutralization of the clustered acidic residue side chains. Protonation of additional residues might further have an impact on the conformation, as suggested by a slightly changed ^15^N-HSQC fingerprint of 3* upon acidification (Fig. 5d).

**Figure 6:**
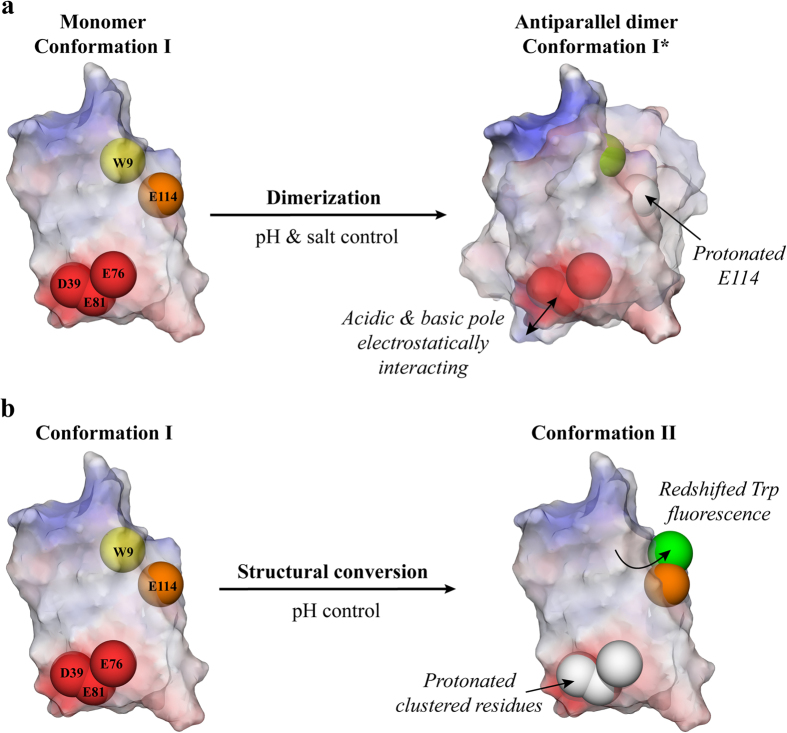
Scheme of pH-induced dimerization and structural conversion of wtNRN1_*L.h.*_ (**a**) Physiological solvent conditions (pH 7.2, NaCl) during spidroin storage cause deprotonated D39, E76, E81 (red spheres) and E114 (orange sphere) to stabilize monomeric NRN1_*L.h.*_ in conformation I. A decreasing NaCl concentration along the spinning duct enables electrostatic interactions between the acidic and basic pole of both subunits, prearranging the dimer in an antiparallel manner. Decreasing ionic strength as well as pH effects residue E114 (orange → white sphere) which induces a slight altered conformation I*, and, thereby, triggers stable dimer formation. (**b**) The structural change into the tight conformation II is initiated simultaneously in the spinning duct but is independently controlled by successive protonation of clustered D39, E76 and E81 (red → white spheres). Thereby, the only W of the domain is shifted towards the surface (yellow → green sphere).

The structural conversion of NRN1_*L.h.*_ was independent of dimerization, and both were controlled by distinct amino acids of the domain. Protonation of E114 apparently triggers the antiparallel dimerization by folding into a slightly altered conformation I*, while the conversion to conformation II is mediated by residues of the acidic cluster (Fig. 6).

## Discussion

The dimerization of the amino-terminal spider silk domain NRN1_*L.h.*_ is triggered by conserved acidic residues in a pH- and salt-dependent manner (Fig. 6). During spidroin storage in the spider’s sac, a neutral pH and the presence of chaotropic salts like NaCl stabilize NRN1_*L.h.*_ in the monomeric conformation I. The decrease of sodium chloride concentration along the silk gland supports the formation of electrostatic interactions between the acidic and basic poles of each monomer, prearranging the antiparallel dimer. Coincident with a dimerization mechanism suggested for NRN1_*E.a.*_^10^, the binding is predominantly stabilized by electrostatic interactions involving residues D39 and E81. Acidification along the spinning duct triggers neutralization of residue E114 and, thereby, slightly changes into conformation I* to strengthen the dimer formation. However, protonation of E76 does not trigger dimerization of NRN1_*L.h.*_ as it was suggested for NRN1_*E.a.*_^10^.

Successive protonation of the structurally neighbouring acidic residues D39, E76 and E81 reduces the charge repulsion between helix 2 and 3 and rearranges the five-helix bundle into the tight conformation II. This mechanism slightly differs from that of NRN1_*E.a.*_ in which apparently only E81 plays a key role^10^. The acidic cluster ensures the correct order of firstly prearranging the dimer by electrostatic interactions and secondly changing the conformation of the dimerized protein.

So far, it is unclear if D39 and E81 get protonated and, thereby, reduce the binding strength of the dimer by breaking intermolecular electrostatic interactions, even so the monomer-dimer distribution remains unaffected at low pH values (Fig. 4a). In accordance with da Silva *et al*. D39 likely has a strong tendency to lose its proton upon dimerization of NRN1_*E.a.*_, but neutralization did not affect dimer formation^44^. In spiders, the pH values were only determined for one species in the first half of the spinning duct due to its extremely small inner diameter making it difficult to speculate about physiologically relevant protonation of residues^11^. In case of further acidification in the second half of the duct its function remains unresolved since a stable NRN dimer is obviously formed at pH 6.0, which is already present in the middle part of the duct.

The sequence of the amino-terminal domain, especially the acidic amino acids at positions 39, 76, 81 and 114, is highly conserved between different spiders^24^. Therefore, it is not surprising that the dimerization mechanism is similar between species. However, the key residues that control the step-by-step dimerization slightly diverge between homologue amino-terminal domains, comparable to discrepancies found between major and minor ampullate spidroins in one species^24^. In case of artificial fiber spinning it will be necessary to identify the molecule-specific tightly controlled process to obtain fibers with a toughness identical to that of natural ones. Since the amino-terminal domain allows to increase the extensibility and strength of fibers made of recombinant spider silk proteins^19^, the stepwise dimerization of their amino-terminal domain is a necessary prerequisite for correct spidroin assembly.

## Additional Information

**How to cite this article**: Bauer, J. *et al*. Acidic Residues Control the Dimerization of the N-terminal Domain of Black Widow Spiders’ Major Ampullate Spidroin 1. *Sci. Rep.* **6**, 34442; doi: 10.1038/srep34442 (2016).

## Supplementary Material

Supplementary Information
