# Supplementary material for: Acidic Residues Control the Dimerization of the N-terminal Domain of Black Widow Spiders’ Major Ampullate Spidroin 1
Source: Sci Rep. 2016 Sep 29;6:34442. doi: 10.1038/srep34442 (PMC5041093; doi:10.1038/srep34442)
Supplement: Supplementary Information [file srep34442-s1.pdf]

Supplementary Information

Acidic Residues Control the Dimerization of the N-terminal Domain of Black Widow Spiders' Major Ampullate Spidroin 1

Joschka Bauer[1], Daniel Schaal[2,3], Lukas Eisoldt[1], Kristian Schweimer[2,3], Stephan Schwarzinger[2,3], and Thomas Scheibel[1,3,4,5,6,*]

[1] Lehrstuhl Biomaterialien, Universität Bayreuth, Fakultät für Ingenieurswissenschaften, Bayreuth, Germany

[2] Lehrstuhl Biopolymere, Universität Bayreuth, Bayrisches Geoinstitut, Bayreuth, Germany

[3] Forschungszentrum für Bio-Makromoleküle (BIOmac), Bayrisches Geoinstitut, Universität Bayreuth, Bayreuth, Germany

[4] Bayreuther Zentrum für Kolloide und Grenzflächen (BZKG), Naturwissenschaften I, Universität Bayreuth, Bayreuth, Germany

[5] Bayreuther Materialzentrum (BayMat), Fakultät für Ingenieurswissenschaften, Universität Bayreuth, Bayreuth, Germany

[6] Bayreuther Zentrum für Molekulare Biowissenschaften (BZMB), Universität Bayreuth, Naturwissenschaften I, Bayreuth, Germany

* Corresponding author

E-mail: thomas.scheibel@bm.uni-bayreuth.de

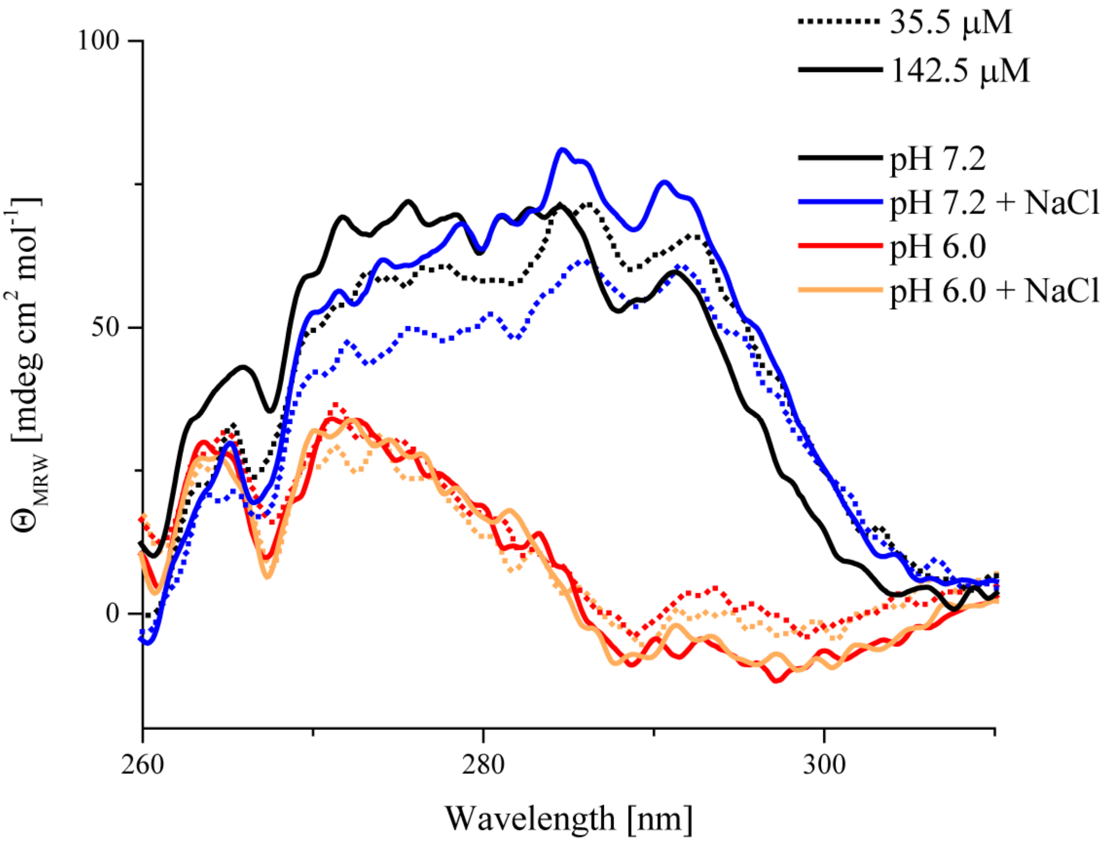

Figure S1. Effect of wtNRN1*L.h.* concentration on near-UV CD spectra. Near-UV CD spectra were taken at a wtNRN1*L.h.* concentration of 142.5 μM using a path length of 0.5 cm (solid line) or at 35.5 μM using a path length of 1.0 cm (dotted). Spectra were measured at pH 7.2 (black), pH 7.2 in the presence of 300 mM NaCl (blue), pH 6.0 (red) and pH 6.0 in the presence of 300 mM NaCl (orange).

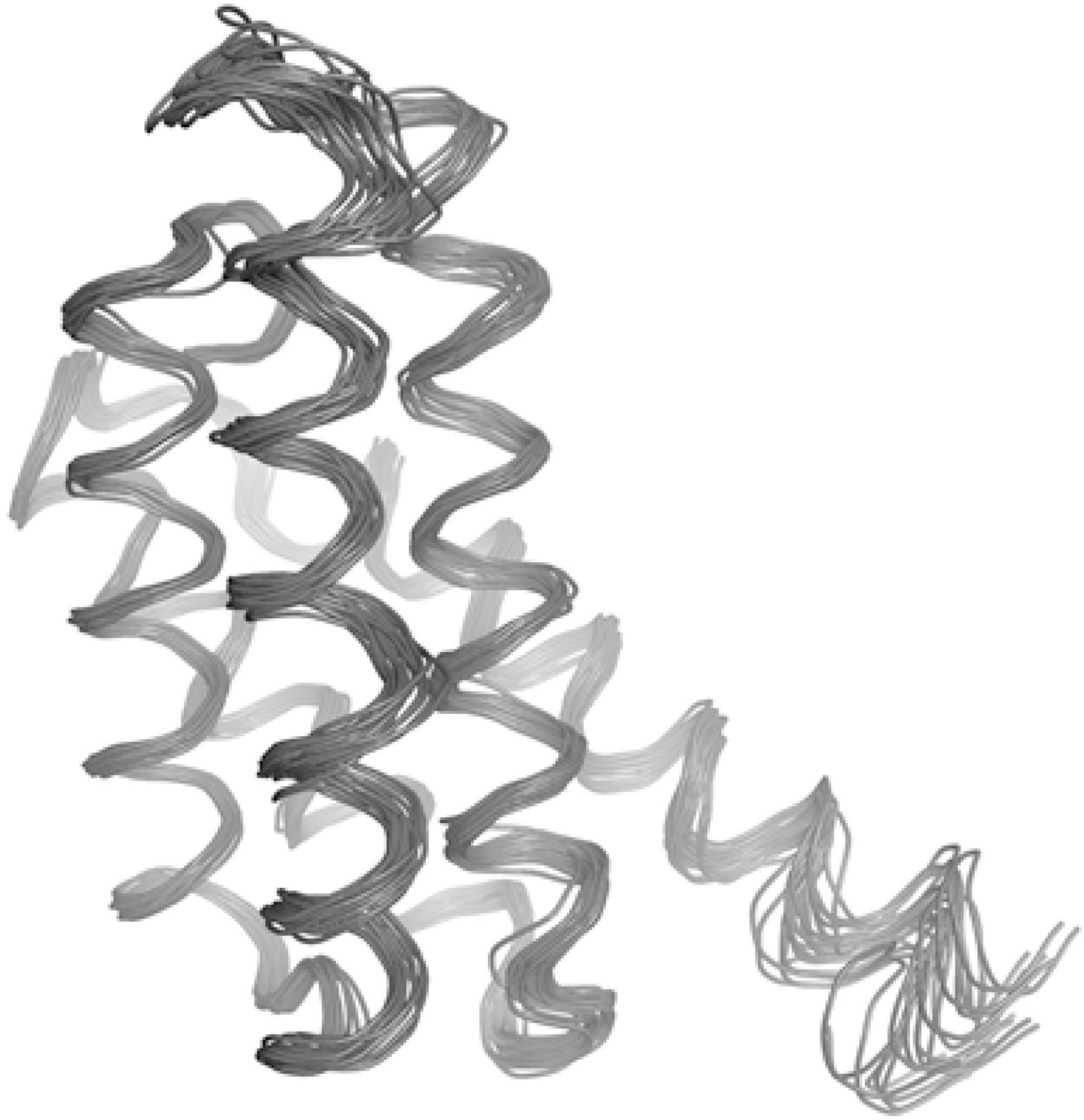

Figure S2. Backbone overlay of the twenty lowest energy structures of NRN1*L.h.* **3*.** The root mean square deviation to the mean structure amounts for 0.53 Å (backbone heavy atoms).

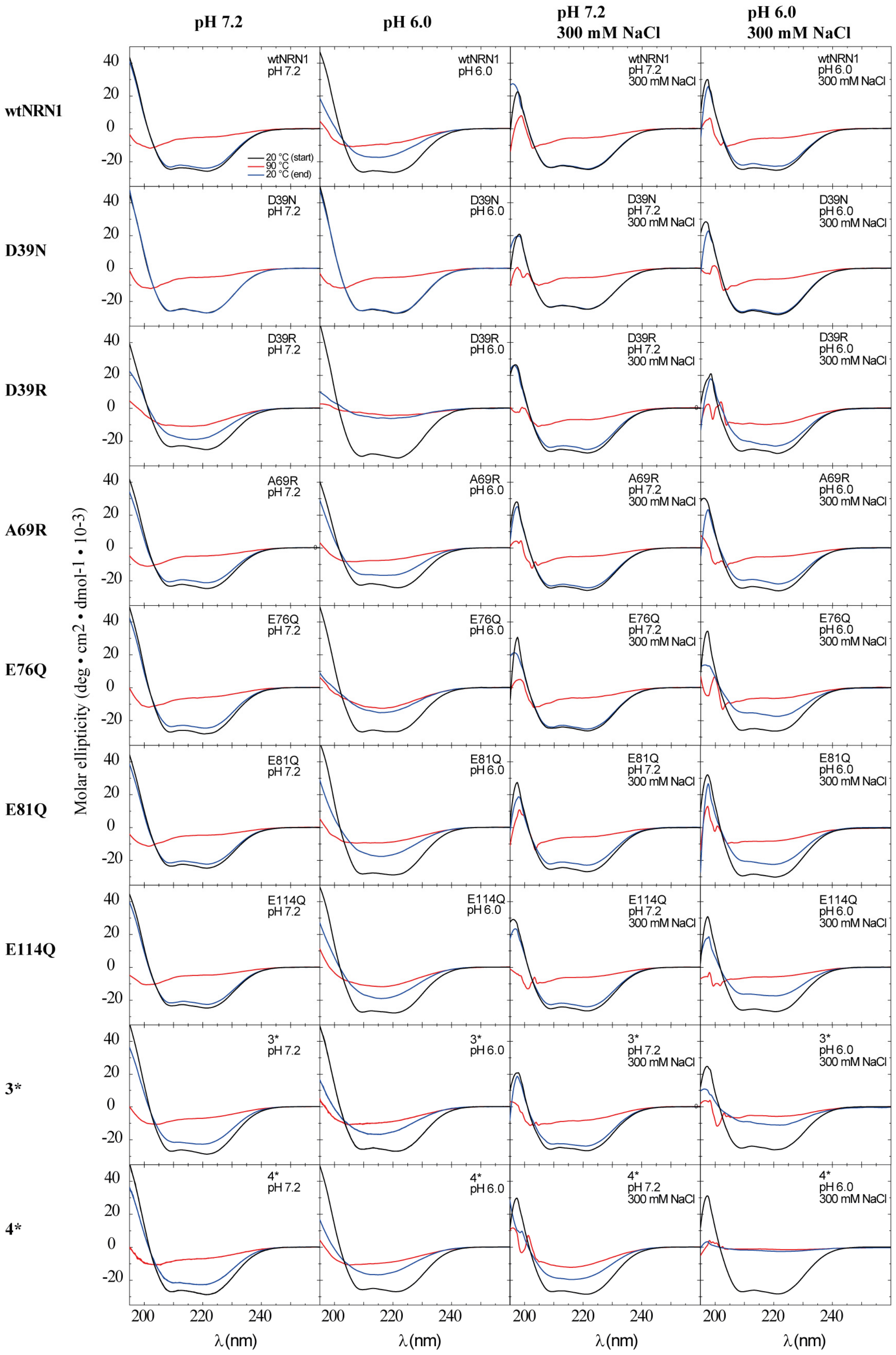

pH 7.2
pH 6.0
pH 7.2 300 mM NaCl
pH 6.0 300 mM NaCl
wtNRN1
D39N
D39R
A69R
E76Q
E81Q
E114Q
3*
4*
20 °C (start)
90 °C
20 °C (end)
Molar ellipticity (deg • cm2 • dmol-1 • 10-3)
λ (nm)

Figure S3. Far-UV CD spectra of NRN1*L.h.* variants. The residual molar ellipticity was initially determined at 20 °C (black line). Afterwards, samples were heated to 90 °C (red) and again cooled to 20 °C (blue).

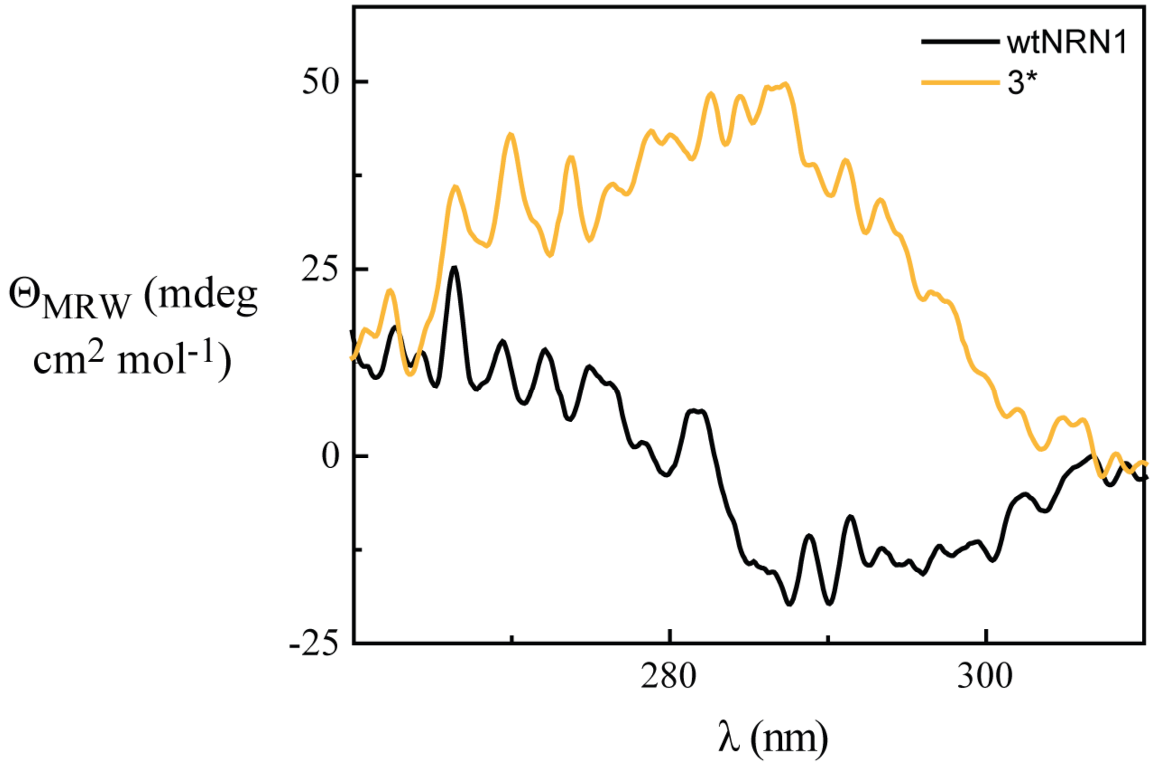

Figure S4. NaCl restricted the structural conversion of variant 3* compared to that of wtNRN1*L.h.*. The difference of the near-UV CD spectra between pH 7.2 and pH 6.0 (see also Figs. 1b and d) in the absence of salt are subtracted by the differential spectra in the presence of 300 mM NaCl (see also Figs. 1c and e). While the presence of NaCl only marginally affected the conformational change of wtNRN1*L.h.*, the structural flexibility of 3* was significantly suppressed.

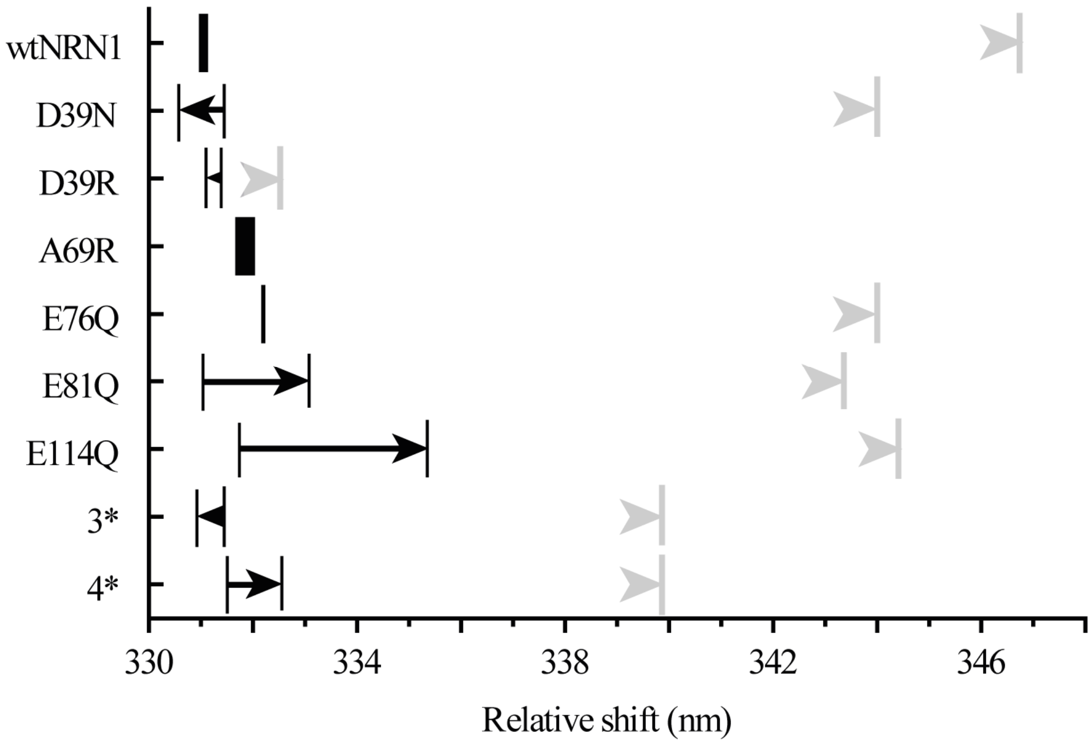

Figure S5. Shift of the fluorescence maximum upon lowering the pH from 7.2 to 6.0 in the presence of sodium chloride. 300 mM NaCl (black arrow) reduces the fluorescence redshift compared to the fluorescence maximum in the absence of salt at pH 7.2 (grey arrow, see also Fig. 2b).

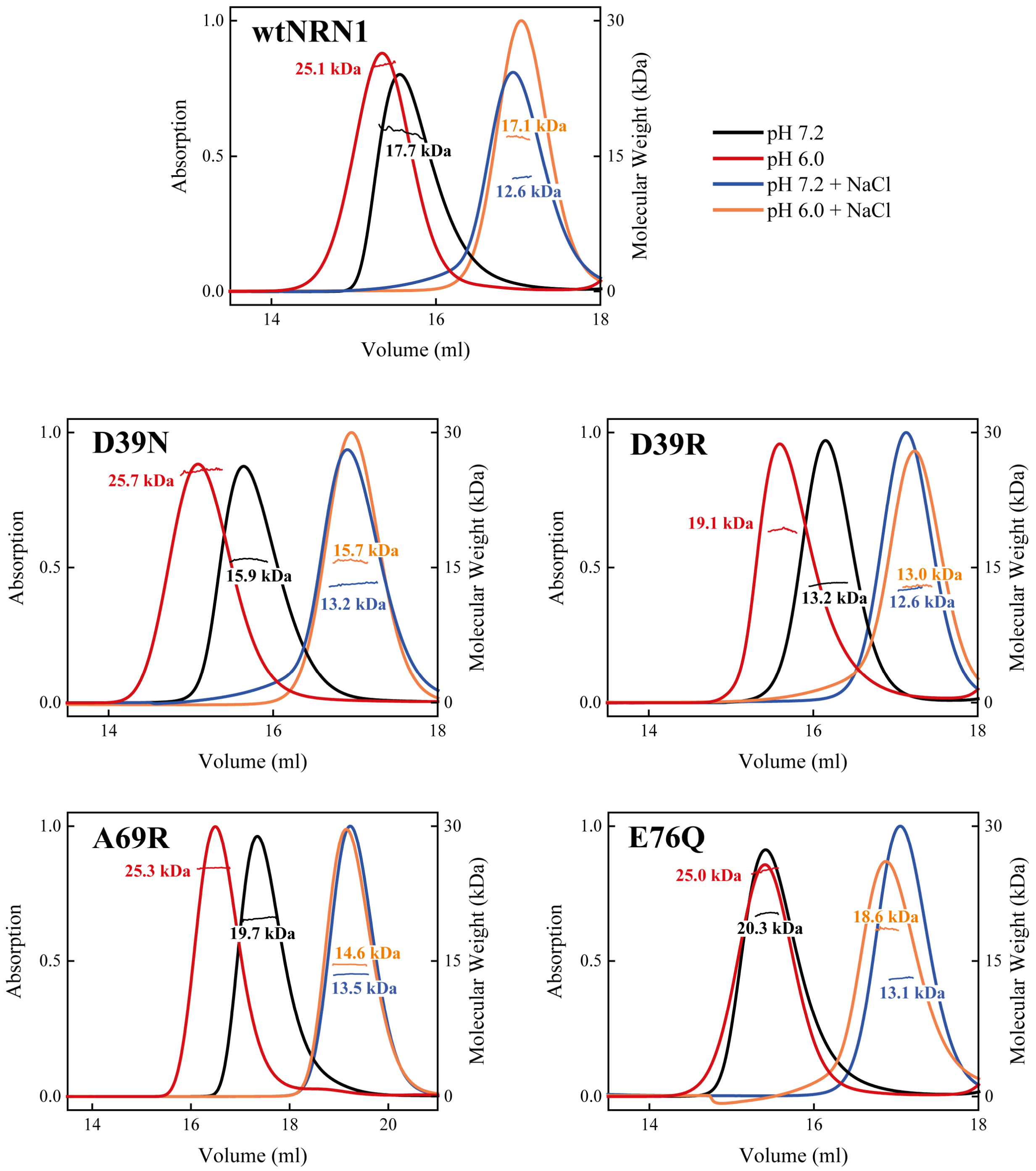

wtNRN1
25.1 kDa
17.7 kDa
17.1 kDa
12.6 kDa
pH 7.2
pH 6.0
pH 7.2 + NaCl
pH 6.0 + NaCl
Absorption
Molecular Weight (kDa)
Volume (ml)
D39N
25.7 kDa
15.9 kDa
15.7 kDa
13.2 kDa
D39R
19.1 kDa
13.2 kDa
13.0 kDa
12.6 kDa
A69R
25.3 kDa
19.7 kDa
14.6 kDa
13.5 kDa
E76Q
25.0 kDa
20.3 kDa
18.6 kDa
13.1 kDa

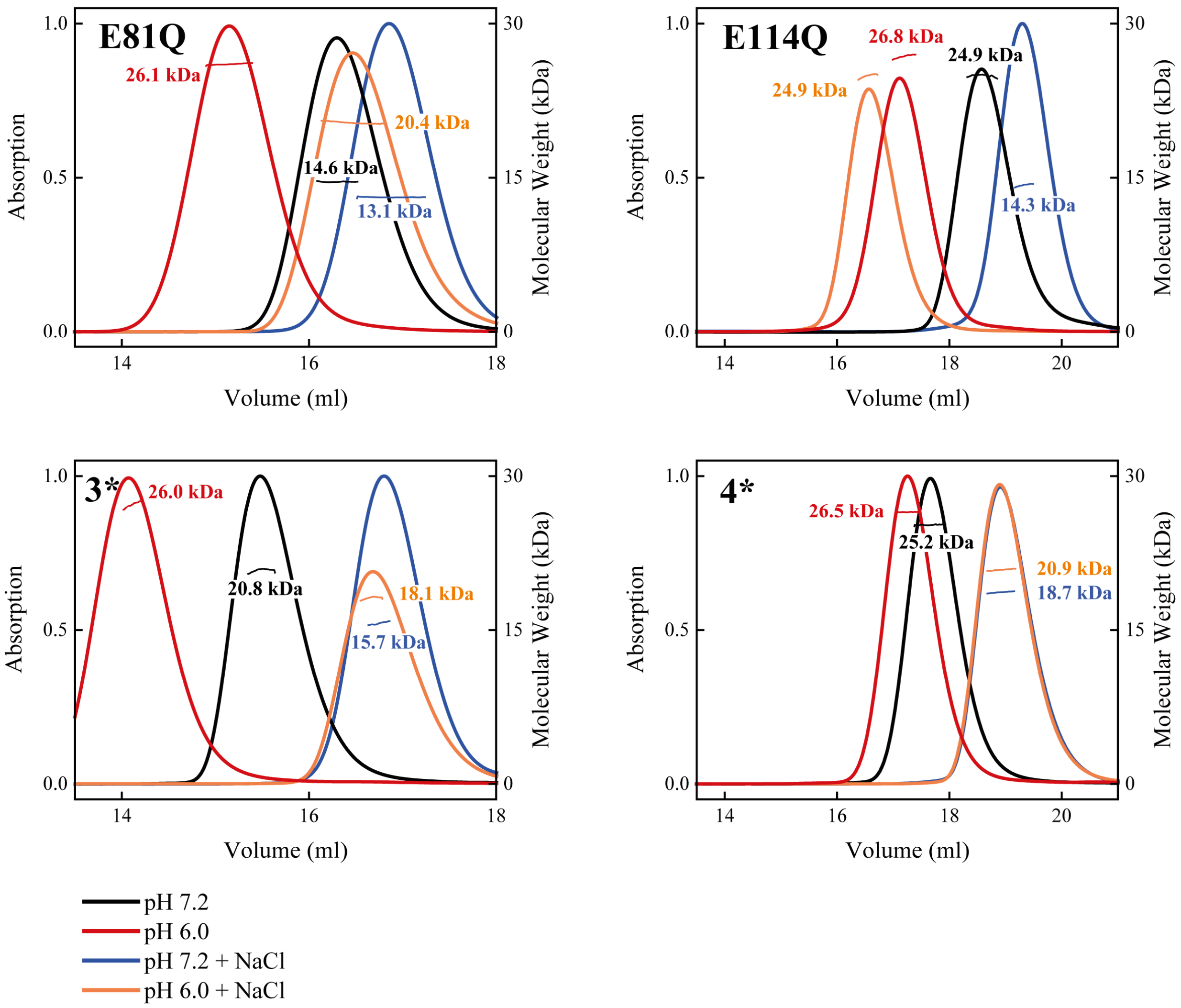

Figure S6. Chromatograms of wildtype as well as variants of NRN1$_{L.h.}$. The chromatograms show the UV signal, and the molar mass of the proteins is indicated. MALS was measured at pH 7.2 (black) as well as pH 6.0 (red) in the absence and presence of 300 mM NaCl (pH 7.2 + NaCl, blue; pH 6.0 + NaCl, orange).

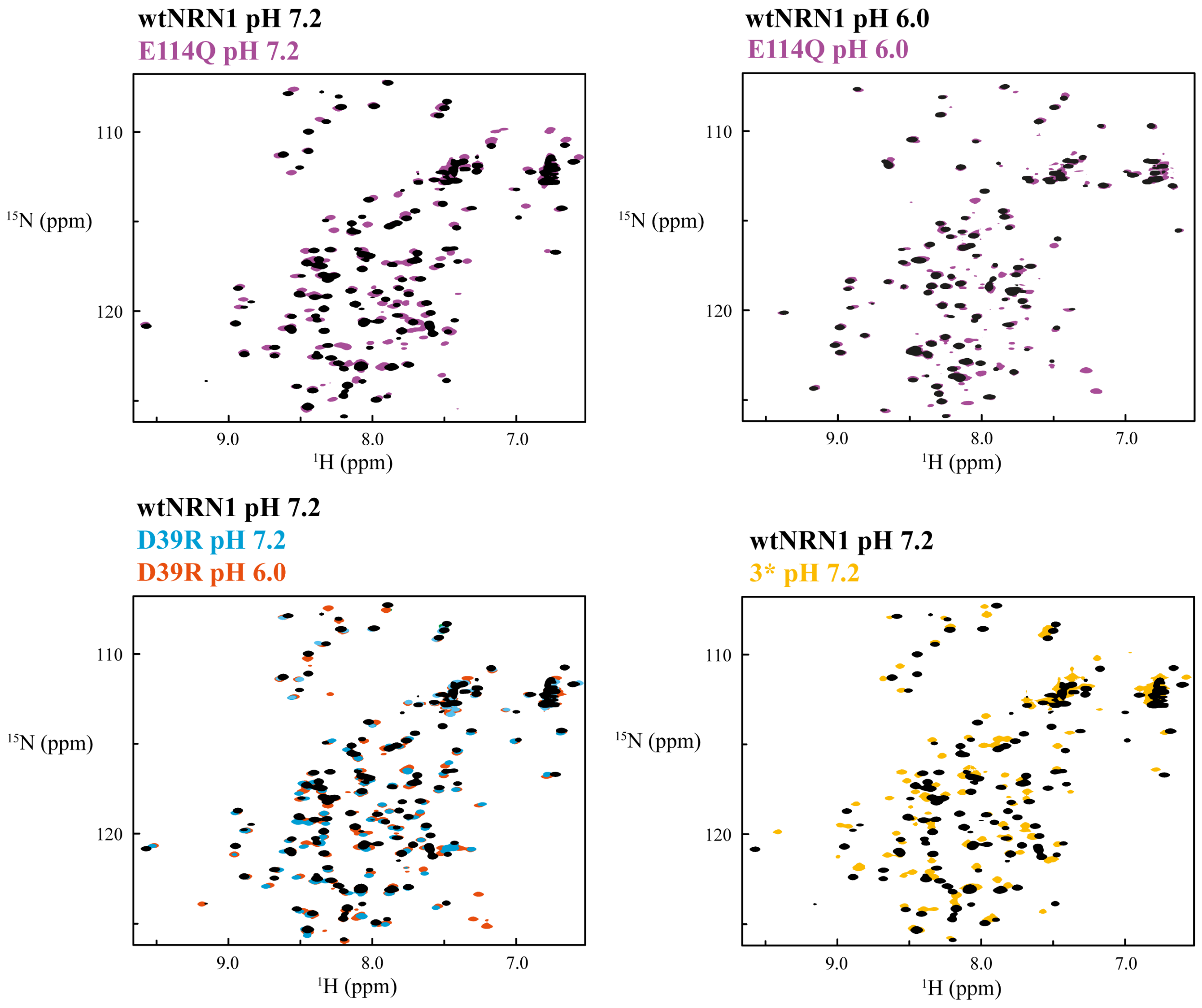

Figure S7: Overlay of HSQC spectra of wtNRN1*L.h.* and variants thereof. For a better comparison of ^{15}N-HSQC fingerprints, the spectrum of wtNRN1*L.h.* (black) was overlaid with spectra of E114Q (purple), D39R at pH 7.2 (blue) and pH 6.0 (red) as well as 3* at pH 7.2 (orange).

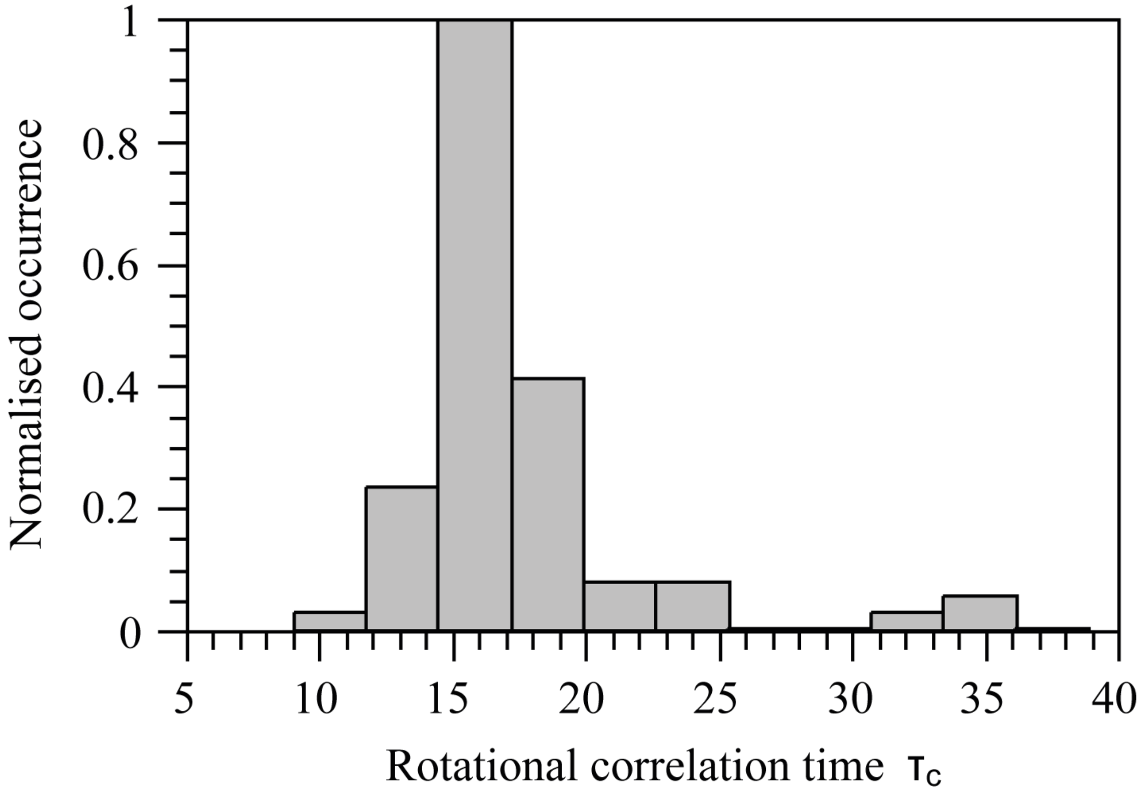

Figure S8. Rotational correlation time τ_c of E114Q at pH 7.2. The rotational correlation time τ_c was estimated using the equation $\tau_c = \sqrt{\frac{6T_1}{T_2} - 7}\frac{1}{4\pi\nu_N}$ with T_1 and T_2 as the longitudinal and transversal relaxation times (delays used in the acquisition of the relaxation data are listed in Supplementary Table S3) and υ_N as the ^{15}N nuclear frequency, respectively[1]. The distribution of the rotational correlation times of the individual sequence positions showed an unimodal pattern with the highest frequency at around 16-17 ns, which is typical for molecular weights of around 25-30 kDa[2], confirming that the variant E114Q dimerizes at a physiological pH.

Table S1. Nuclear overhauser effect (NOE) restraints and structural statistics for the best 20 structures of NRN1$_{L.h.}$ variant 3*.

**Experimental derived restraints**		
distance restraints	NOE	1253
	intraresidual	9
	sequential	466
	medium range	413
	long range	365
dihedral restraints		224
**restraint violation**		
average distance restraint violation (Å)		0.008118 (+/- 0.000900)
distance restraint violation > 0.1 Å		0.95 (+/- 1.20)
distance restraint violation > 0.3 Å		0.00 (+/- 0.00)
average dihedral restraint violation (°)		0.3799 (+/- 0.0332)
dihedral restraint violation > 1°		8.45 (+/- 1.63)
dihedral restraint violation > 3°		0.05 (+/- 0.22)
dihedral restraint violation > 5°		0.00 (+/- 0.00)
**deviation from ideal geometry**		
bond length (Å)		0.000863 (+/- 0.000058)
bond angle (°)		0.1715 (+/- 0.0084)
**coordinate precision**[a,b]		
backbone heavy atoms (Å)		0.53
all heavy atoms (Å)		0.89
**Ramachandran plot statistics**[c] (%)	regions	
	most favoured	93.3
	additionally allowed	6.2
	generously allowed	0.4
	disallowed	0.0

[a] The precision of the coordinates is defined as the average atomic root mean square difference between the accepted simulated annealing structures and the corresponding mean structure calculated for the given sequence regions.
[b] calculated for residues Ala5-Ala127
[c] Ramachandran plot statistics are determined by PROCHECK (Laskowski, et al., 1993) and noted by most favored/additionally allowed/generously allowed/disallowed.

Table S2. Stability of NRN1*L.h.* variants. Protein denaturation was analysed as a function of urea concentration or temperature using CD ellipticity at 222 nm. The urea concentration and melting temperature (T_m) refer to the condition at which half of the proteins are denatured. The min/max values at distinct solvent conditions are highlighted in blue/red, and equally stable variants are marked by stars (*).

	**Urea (M)**		**T_m (°C)**			
	pH 7.2	pH 6.0	pH 7.2	pH 7.2 + 300 mM NaCl	pH 6.0	pH 6.0 + 300 mM NaCl
**wtNRN1**	1.8	1.9	47.4	59.8	65.5*	62.1
**D39N**	2.9	3.0	53.3	63.3	54.2	63.5
**D39R**	4.2*	4.4*	61.2	71.3	65.6*	69.8
**A69R**	1.7	2.3	42.4	54.9	45.8	57.2
**E76Q**	2.4	2.4	55.0	62.9	65.6*	64.8
**E81Q**	2.4	2.4	49.3	59.8	62.6	61.5
**E114Q**	2.4	2.7	63.0	60.3	64.9	63.0
**3***	3.5	3.4	60.2	65.7	61.0	67.3
**4***	3.7	3.8	64.7	65.1	62.7	68.4

Table S3. ***T_1*** **and** ***T_2*** **relaxation delays used in the acquisition of** **^{15}N-HSQC spectra of variant E114Q.**

T_2 relaxation delays (ms)	T_1 relaxation delays (ms)
8.48	10.642
8.48	10.642
16.96	265.57
25.44	265.57
25.44	637.34
33.92	637.34
42.4	1,062.22
42.4	1,062.22
50.88	1,380.88
59.36	1,380.88
59.36	1,911.98
67.84	1,911.98
84.8	2,655.52
84.8	2,655.52
93.28	
93.28	

References

1 Kay, L. E., Torchia, D. A. & Bax, A. Backbone Dynamics of Proteins as Studied by N-15 Inverse Detected Heteronuclear Nmr-Spectroscopy - Application to Staphylococcal Nuclease. *Biochemistry* **28**, 8972-8979, (1989).

2 Rossi, P. *et al.* A microscale protein NMR sample screening pipeline. *J. Biomol. NMR* **46**, 11-22, (2010).
